# Fabrication of Highly Transparent Y_2_O_3_ Ceramics with CaO as Sintering Aid

**DOI:** 10.3390/ma14020444

**Published:** 2021-01-18

**Authors:** Danlei Yin, Jun Wang, Meng Ni, Peng Liu, Zhili Dong, Dingyuan Tang

**Affiliations:** 1 Jiangsu Key Laboratory of Advanced Laser Materials and Devices, School of Physics and Electronic Engineering, Jiangsu Normal University, Xuzhou 221116, China; yi0001ei@e.ntu.edu.sg (D.Y.); liupeng_jsnu@jsnu.edu.cn (P.L.); 2 School of Electrical and Electronic Engineering, Nanyang Technological University, Singapore 639798, Singapore; nime0003@e.ntu.edu.sg; 3 School of Materials Science and Engineering, Nanyang Technological University, Singapore 639798, Singapore; zldong@ntu.edu.sg

**Keywords:** yttrium oxide, transparent ceramics, sintering aids

## Abstract

Highly transparent Y_2_O_3_ ceramics were successfully fabricated with CaO as sintering aid. The microstructure evolution, optical transmittance, hardness and thermal conductivity of the Y_2_O_3_ ceramics were investigated. It was found that doping a small amount (0.01–0.15 wt.%) of CaO could greatly improve the densification rate of Y_2_O_3_. With an optimized CaO dosage of 0.02 wt.% combined with the low temperature vacuum sintering plus hot isostatic pressing (HIP-ing), Y_2_O_3_ ceramics with in-line transmittance of 84.87% at 1200 nm and 81.4% at 600 nm were obtained.

## 1. Introduction

Due to their unique optical and thermal properties such as high thermal conductivity, low thermal expansion coefficient, low phonon energy, and low infrared emissivity at elevated temperature [1,2,3,4,5], transparent Y_2_O_3_ ceramics have a number of important applications, e.g., they are considered as a promising host material for high efficiency solid-state lasers, they can be used as supersonic infrared windows and missile domes. However, partly owing to the high melting temperature of Y_2_O_3_ (2430 °C), it is hard to fabricate highly transparent Y_2_O_3_ ceramics, especially at a relatively low sintering temperature [6].

It is widely known that intragranular pores can be generated and remained after sintering. In order to promote densification rate and avoid the formation of intragranular pores, cation ions with different valences are usually utilized as sintering aids. In previous studies, tetravalent sintering aids, for example, Th^4+^, Hf^4+^, and Ti^4+^ were reported [7,8,9]. Zr^4+^ as a sintering aid was widely studied, and highly transparent Zr-doped Y_2_O_3_ ceramics were fabricated [10,11,12]. Trivalent cations like La^3+^ were also proven to be an effective sintering additive for Y_2_O_3_ in a number of studies [13,14,15,16]. A few studies were conducted using divalent ions, such as Ca^2+^ and Mg^2+^, as sintering aids. In 1990, Katayama et al. first reported the sintering and electrical properties of 1 mol% CaO doped Y_2_O_3_ sintered in air and proved that CaO was effective in improving the sinterability [17,18]. In 2001, the diffusion mechanism of defects in Ca-doped Y_2_O_3_ ceramics was studied by Saito et al. [19]. In 2010, Kodo et al. investigated the grain boundary mobility of divalent cation doped Y_2_O_3_ that were pressurelessly sintered in air with a doping concentration of 1 mol% [20]. However, as the proper doping concentration of CaO in Y_2_O_3_ was not investigated in the previous studies, no highly transparent Y_2_O_3_ ceramics with CaO as sintering aid were fabricated. 

In the present work, we investigated Y_2_O_3_ transparent ceramics fabrication using CaO as sintering aid. Highly transparent Y_2_O_3_ ceramics was successfully fabricated using vacuum sintering followed by hot isostatic pressing (HIP) technique. We report on the sintering behaviors of the Y_2_O_3_ samples under different CaO doping concentrations, and the optical, mechanical, and thermal properties of the as-sintered ceramics. 

## 2. Experimental Procedure

### 2.1. Ceramic Fabrication

The starting materials were commercial Y_2_O_3_ powder (99.999%, Jiahua Advanced Material Resources Co., Ltd., Jiangyin, China) and calcium oxide powder (CaO, 99.99%, Sigma-Aldrich, St. Louis, MO, USA). They were mixed together with the ratio of 0.01 wt.%, 0.02 wt.%, 0.05 wt.% and 0.15 wt.% of CaO, respectively. The mixed powders were ball milled for 15 h using ethanol as ball-milling media. After drying and sieving, the obtained powders were calcined at 1200 °C for 5 h and pressed into 20 mm diameter compacts under a manual tablet press machine. The compacts were then cold isostatic pressed (CIP-ed) under 200 MPa. The green bodies were first vacuum sintered (MT-U-1822, Meiteng, Suzhou, China) at a temperature in the range of 1450–1650 °C. After that, the Y_2_O_3_ ceramics were hot isostatic pressed (10–30H, AIP, Columbus, OH, USA) at 1510 °C under 196 MPa, then annealed and polished into 3 mm in thickness.

### 2.2. Characterizations

The relative density was measured by the Archimedes method. The average grain size was measured by the software called Nano Measure, taking at least 200 grains into account for each sample. The microstructure of the as-sintered ceramics was observed using a scanning electron microscopy (JSM-6360A, JEOL, Tokyo, Japan). The in-line transmission was characterized by a UV-VIS-NIR spectrometer (Lambda 950, Perkin Elmer, Waltham, MA, USA). The Vickers hardness was measured by using a microhardness tester (FM-300e, FUTURE-TECH, Kanagawa, Japan), with the applied load of 1 kg. The thermal diffusivity was measured by the laser-flash method (DLF 1200, TA Instruments, New Castle, DE, USA).

## 3. Results and Discussion

**Figure 1:**
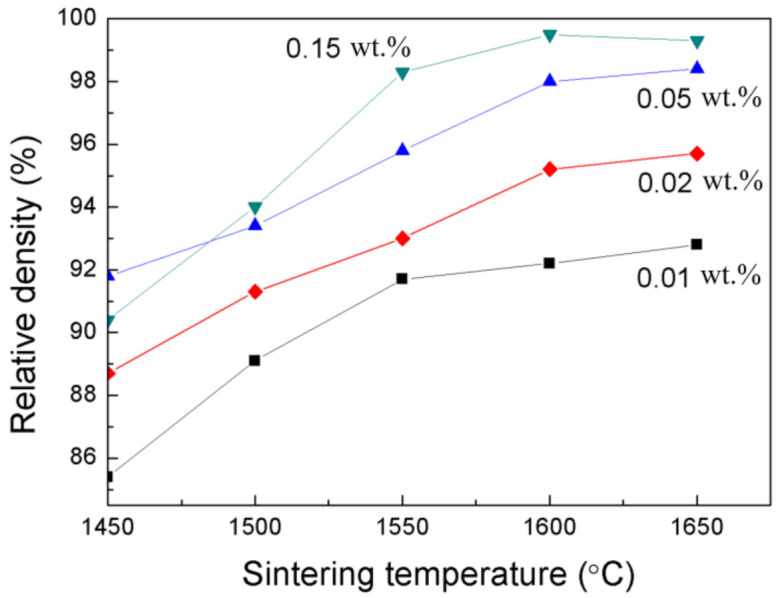
Relative density versus vacuum sintering temperature of the Y_2_O_3_ samples with different CaO contents.

Figure 1 shows the relative density of Y_2_O_3_ samples with different CaO doping concentrations and vacuum sintered at 1450–1650 °C. At the same sintering temperature, the relative density of the samples increased as the CaO content increased. The maximum density that could be reached also increased with the CaO doping concentration. For example, the 0.15 wt.% CaO-doped Y_2_O_3_ reached its maximum relative density (99.5%) at 1600 °C, while the highest density of the 0.01 wt.% CaO-doped Y_2_O_3_ was merely 92.8%. The densification rate obviously slowed down after 1550 °C, which indicates that grain growth rather than volume shrinkage is dominant in the stage. It is important to note that there was only slight density increase for the 0.01 wt.% CaO-doped sample even when the sintering temperature was further increased.

**Figure 2:**
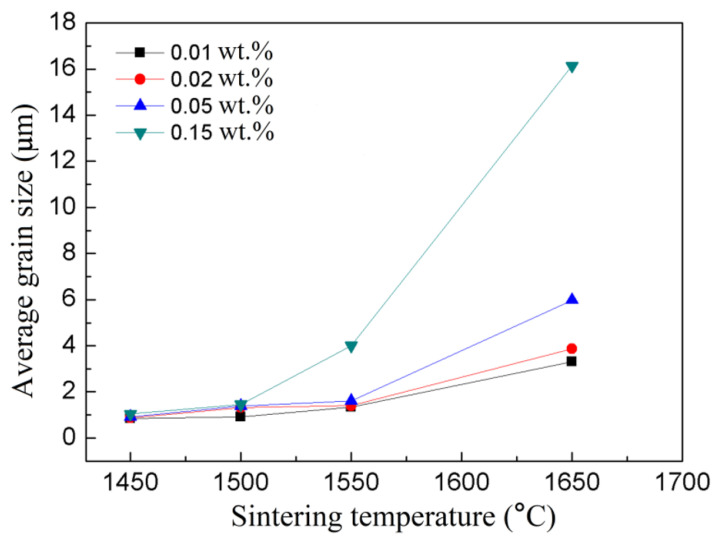
Average grain size versus vacuum sintering temperature of Y_2_O_3_ samples with different CaO contents.

Figure 2 shows the variation of average grain size as a function of sintering temperature of the CaO-doped Y_2_O_3_ ceramics. At a given sintering temperature, the samples with higher doping concentration show higher grain growth rate and exhibit larger average grain size. It indicates that adding CaO has obviously accelerated the rate of grain boundary migration. What’s more, the influence on the grain boundary mobility tends to be greater with higher doping concentration. This phenomenon could be explained by the following defect analysis.

As reported previously, the introduction of calcium oxide into yttria under high oxygen pressures can be described as: [17]

(1) $$2\mathrm{CaO}+\frac{1}{2}O_{2}\left(g\right)⇌{2\mathrm{Ca}}_{Y}^{\prime }{+3O}_{O}{+2h}^{\cdot }$$

where the standard notations of Kröger and Vink were used here [21]. Ca_Y_^’^ and O_O_ are a Ca^2+^ ion on an yttria site and an O^2−^ ion on its regular site, respectively, and h is an electron hole. Considering the vacuum sintering condition in this experiment, where the oxygen pressure is negative, the oxygen vacancies are easily generated according to Equation (2):

(2) $$O_{O}⇌V_{Ö}+\frac{1}{2}O_{2}↑{+2e}^{\prime }$$

Thus, the real reaction under the present experimental conditions can be expressed as:

(3) $$2\mathrm{CaO}⇌{2\mathrm{Ca}}_{Y}^{\prime }{+V}_{Ö}{+2O}_{O}$$

Owing to the similar ionic radius of Ca^2+^ (1.12 Å) and Y^3+^ (1.02 Å), Ca^2+^ could substitute the site of Y^3+^, creating one negative charge. Therefore, by adding Ca^2+^ into Y_2_O_3_ under vacuum sintering condition, additional oxygen vacancies could be created. The increased concentration of CaO drives the equilibrium (3) to the right side, which contributes to the concentration increase of oxygen vacancies. Chen et al. have demonstrated that the grain boundary mobility in Y_2_O_3_ is dominated by cation diffusivity, which can be enhanced by the presence of oxygen vacancies [14]. Thus, the grain boundary mobility could be promoted by the introduction of CaO.

**Figure 3:**
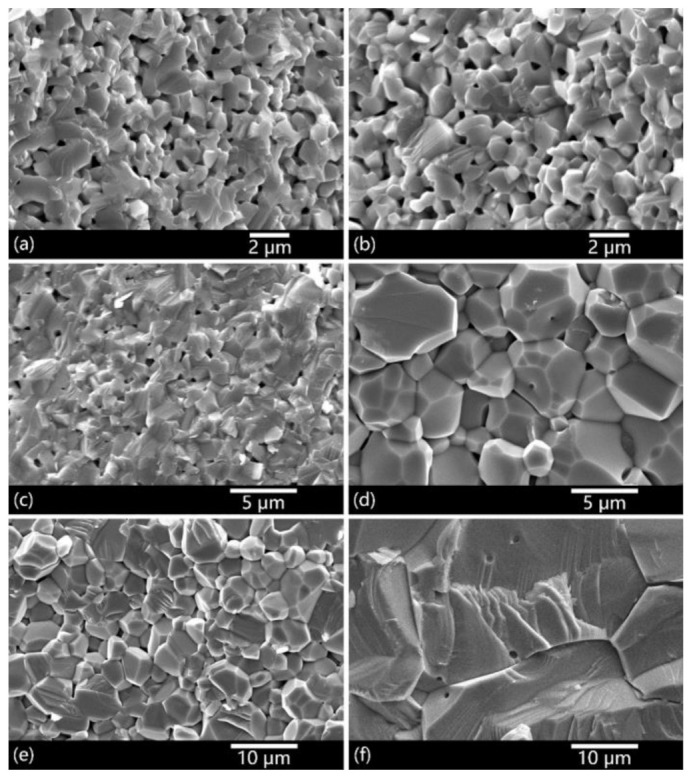
SEM images of 0.02 wt.% CaO-doped (**a**,**c**,**e**) and 0.15 wt.% CaO-doped samples (**b**,**d**,**f**), vacuum sintered at 1450 °C, 1550 °C and 1650 °C, respectively.

Figure 3 shows the microstructure evolution of the 0.02 wt.% and 0.15 wt.% CaO-doped samples under different sintering temperatures. At 1450 °C, both the 0.02 wt.% and 0.15 wt.% samples (Figure 3a,b) have the same degree of porosity and grain size (~0.8 μm). As the temperature was increased, the 0.15 wt.% CaO-doped samples showed an obviously faster densification and grain growth rate, which is consistent with the densification curve shown in Figure 1. At 1650 °C, the average grain size of the 0.15 wt.% CaO-doped sample had increased to over 10 μm, with a certain number of intragranular pores. In contrast, intragranular pores were not detected in the 0.02 wt.% CaO-doped samples. It means grain coarsening, rather than densification, has played the leading role in the CaO heavily doped samples. That is why for the 0.15 wt.% CaO-doped samples there was a slight density decrease in Figure 1 as the temperature was further increased from 1600 °C to 1650 °C. Therefore, further densification was difficult in the 0.15 wt.% CaO-doped samples.

**Figure 4:**
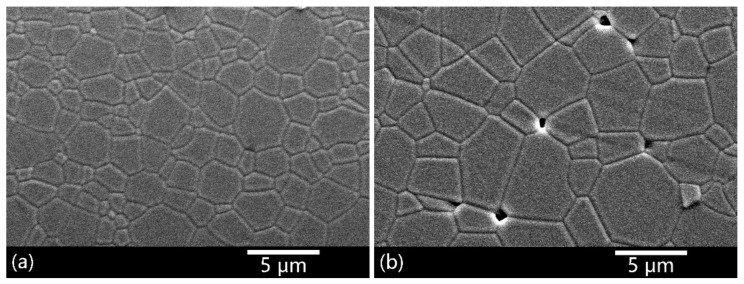
SEM images of the ceramics after vacuum sintering 1550 °C and HIP-ing at 1510 °C with CaO concentration of (**a**) 0.02 wt.% and (**b**) 0.15 wt.%.

Figure 4 shows the SEM images of the ceramics after vacuum sintering at 1550 °C followed by HIP-ing at 1510 °C. After HIP-ing, the 0.02 wt.% CaO-doped ceramics exhibited a fully densified structure, without the detection of residual pores and secondary phases. Relative density of the samples achieved above 99.99%. In contrast, relative density of the ceramic shown in Figure 4b was just 99.8%. It is known that the driving force for densification decreases with the increase of grain size. In addition, during HIP, the plastic deformation mechanism is weaker in coarser grained samples. That is the reason why the pores in the 0.15 wt.% CaO-doped ceramics were more difficult to remove.

**Figure 5:**
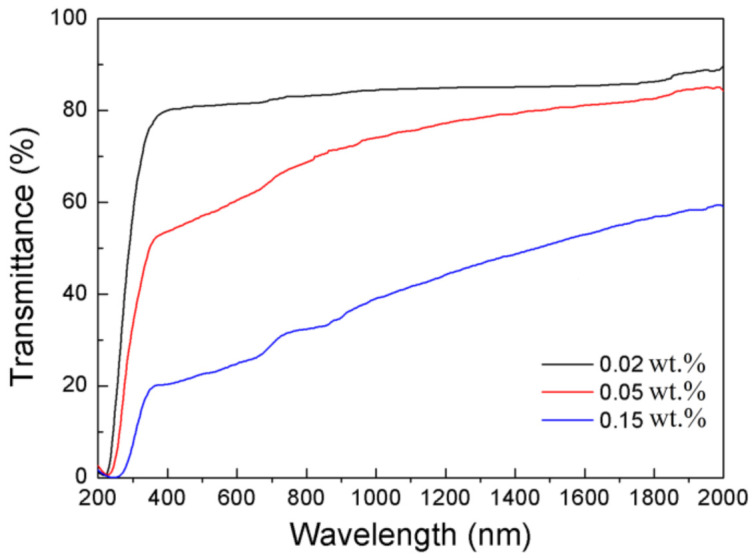
In-line transmission of the Y_2_O_3_ ceramics with different CaO contents vacuum sintered at 1550 °C and HIP-ed at 1510 °C.

Figure 5 shows the in-line transmission of the CaO-doped Y_2_O_3_ ceramics after the HIP-ing, with a thickness of 3 mm. The 0.01 wt.% CaO-doped Y_2_O_3_ ceramic was completely opaque, whose transmission line is zero throughout the whole wavelength range. The 0.02 wt.% CaO-doped Y_2_O_3_ showed the highest in-line transmission over a wavelength range of 0.2–2 μm, reaching 84.87% at 1200 nm and remaining 81.41% at 600 nm. The transmission degraded when the CaO doping concentration was increased. It reveals that the optical property of the final sintered samples was very sensitive to the CaO doping concentration. Under the present sintering condition, 0.02 wt.% could be a favorable doping concentration. It demonstrates that residual pores could be effectively eliminated by adding a small amount of CaO to accelerate the densification during sintering process. As far as we know, highly transparent Y_2_O_3_ ceramics with CaO as sintering additive have never been reported before.

The Vickers hardness of the Y_2_O_3_ ceramics vacuum sintered at 1550 °C followed by HIP-ing at 1510 °C was measured and is shown in Table 1. Generally, the Vickers hardness decreased with increasing CaO content. For example, the Vickers hardness of the undoped Y_2_O_3_ ceramics, initially 738.72, decreased to 689.06 as the doping concentration reached 0.15 wt.%. 

**Figure 6:**
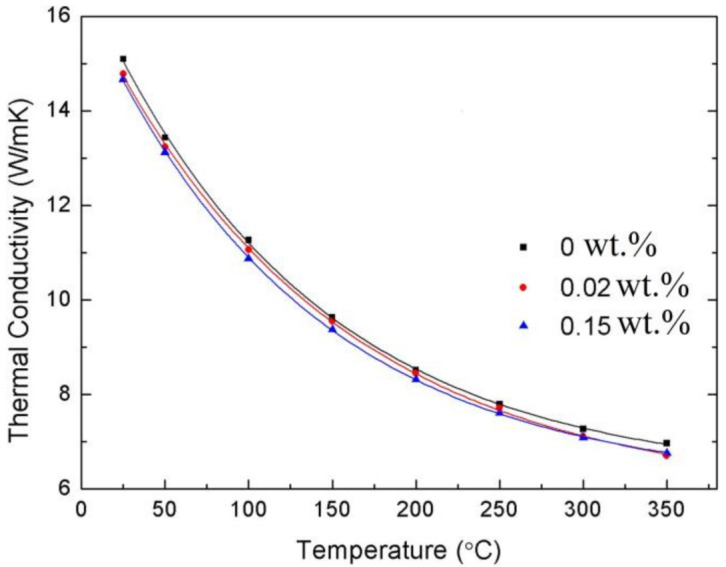
Thermal conductivity of the Y_2_O_3_ ceramics with different contents of CaO versus temperature.

The thermal conductivity of the transparent Y_2_O_3_ ceramics with different contents of CaO is further studied, as shown in Figure 6. It is calculated according to the formula:

(4) $$\lambda_{C}=\alpha \rho C$$

where α is the thermal diffusivity measured by the laser-flash method, ρ is the theoretical density of Y_2_O_3_ (5.01 g/cm^3^), and C is the specific heat capacity of Y_2_O_3_ calculated by [22]:

(5) $$C\left(J/g\cdot K\right)=0.441+1.284\times 10^{-4}\times T$$

In comparison with the undoped Y_2_O_3_ sample, there is a slight decrease of the thermal conductivity with increasing CaO doping concentration. This is reasonable because the mismatch of the atomic mass and ionic radius between the substitutions and host crystalline materials could lead to phonon scattering and thermal conductivity reduction [23]. However, the degradation of the thermal conductivity was very small owing to the small amount of CaO doping. For example, the thermal conductivity of 0.15 wt.% CaO-doped sample still reached 14.6 W/mK at room temperature.

## 4. Conclusions

In conclusion, using CaO as sintering aid, we have successfully fabricated highly transparent Y_2_O_3_ ceramics by low temperature vacuum sintering plus HIP-ing. The effects of CaO doping concentration on the sintering behavior, optical transmission, mechanical strength and thermal properties of Y_2_O_3_ were investigated. It is shown that even a small amount of variation in CaO doping could effectively affect Y_2_O_3_ sinterability and the exclusion of residual pores inside the ceramics. The highest in-line transmission (81.41% at 600 nm) was achieved on samples with 0.02 wt.% CaO-doping, vacuum sintered at 1550 °C and HIP-ed at 1510 °C. The samples have Vickers hardness of 723.84 HV and thermal conductivity at room temperature of 14.8 W/mK.

## Figures and Tables

**Table 1:** Mechanical property of Y_2_O_3_ ceramics with different CaO content.

CaO Content (wt.%)	Vickers Hardness (HV)
0	738.72
0.01	722.21
0.02	723.84
0.05	706.14
0.15	689.06

## Data Availability

The data presented in this study are available on request from the corresponding authors.
